# The median effective analgesic concentration of ropivacaine in ultrasound-guided interscalene brachial plexus block after arthroscopic rotator cuff repair

**DOI:** 10.3389/fphar.2022.928227

**Published:** 2022-08-17

**Authors:** Cheng Xu, Fei Gu, Yang Liu, Rui Chen, Chengyu Wang, Jie Lu

**Affiliations:** Department of Anaesthesiology, Shanghai Jiaotong University Affiliated Sixth People’s Hospital, Shanghai, China

**Keywords:** interscalene brachial plexus block, median effective analgesic concentration, postoperative analgesia, arthroscopic rotator cuff repair, ropivacaine

## Abstract

**Background:** The median effective analgesic concentration (MEAC) of ropivacaine in interscalene brachial plexus block (ISBPB) for postoperative analgesia after arthroscopic rotator cuff repair (ARCR) has not been determined. Therefore, this study aimed to evaluate the MEAC after ARCR using 10 ml ropivacaine.

**Method:** This study was conducted on 40 patients with American Society of Anesthesiologists grade I or II who had selective ARCR. The 10 ml ropivacaine was administered for determined, with an initial concentration of 0.3% using up-and-down sequential allocation. After successful or unsuccessful postoperative analgesia, the concentration of ropivacaine was decreased or increased by 0.05% in the next patient. We defined successful postoperative analgesia as a visual analog scale score of <4 at rest within the initial 8 h after ISBPB. The analytic techniques of linear, linear-logarithmic, exponential regressions and centered isotonic regression were used for calculating MEAC. The secondary outcomes was sufentanil consumption, time to 1st rescue analgesic, onset time of sensory block and motor block.

**Results:** The concentration of ropivacaine administered ranged from 0.1% to 0.35%. The MEAC from the four different methods (linear, linear-logarithmic, exponential regressions and centered isotonic regression) were 0.207% (95% CI, 0.168–0.355%), 0.182% (95% CI, 0.165–0.353%), 0.196% (95% CI, 0.154–0.356%), and 0.163%, respectively. Of the four models, exponential regression had the least residual standard error (0.0990).

**Conclusion:** The MEAC derived from the four statistical models for 10 ml ropivacaine in ultrasound-guided ISBPB for postoperative analgesia was distributed within a narrow range of 0.163%–0.207%. The exponential regression model calculated by the goodness-of-fit test at a concentration of 0.196% best fits the study data.

**Clinical Trial Registration:** http://www.chictr.org.cn/showproj.aspx?proj=127449, identifier ChiCTR2100047978

## 1 Introduction

Arthroscopic rotator cuff repair (ARCR) usually results in moderate to severe pain (Neuts et al., 2018), and the interscalene brachial plexus block (ISBPB) is still considered the most effective analgesic strategy during ARCR (Dhir et al., 2016; Warrender et al., 2017; Rhyner et al., 2020). Ropivacaine is the most common long-acting local anesthetic for ISBPB in clinical practice. However, numerous adverse effects can occur (e.g. hypotension, motor blockade, phrenic nerve paralysis, and poisoning reactions) when high doses of ropivacaine are used (Grelet et al., 2021).

The appropriate volume and concentration of local anesthetic are crucial in reducing the adverse effects of peripheral nerve blocks (Fredrickson et al., 2010). Low concentrations of ropivacaine can achieve similar analgesic effects as high concentrations while reducing the incidence of adverse reactions associated with local anesthetics and minimizing motor blockade (Borgeat et al., 2010; Christiansen et al., 2018). Patients undergoing ARCR are encouraged to perform functional exercises of the upper limbs postoperatively. Therefore, finding an appropriate concentration of ropivacaine in ISBPB that ensures both analgesia and reduced motor blockade is potentially beneficial for rapid recovery after ARCR.

The ultrasound-guided nerve block technique has many advantages, such as accurate nerve localization and the precise identification of the injection needle tip. This visualization technique potentially reduces the total amount of local anesthetic applied and reduces the risk of toxic reactions (Marhofer et al., 2010).

However, no data are available regarding the minimum effective analgesic concentration (MEAC) of ropivacaine for performing ISBPB during the ARCR. Therefore, the primary objective of this study was to evaluate the MEAC (EC50 = effective concentration in 50% of patients) after ARCR using 10 ml ropivacaine.

## 2 Methods

### 2.1 Study design and population

This single-arm prospective double-blind study was approved by the Ethics Committee of Shanghai Jiaotong University Affiliated Sixth People’s Hospital (reference No. 2021–145) and registered with the Clinical Trial Registry of China (ChiCTR2100047978). All subjects underwent inpatient surgical treatment and signed the informed consent form.

The study was conducted in Shanghai Jiaotong University Affiliated Sixth People’s Hospital. The same team of surgeons performed the surgical procedures. An anesthetic assistant conducted an anesthetic visit and assessment the day before surgery to register and recruit subjects who met the inclusion criteria. Patients were included in the study if they had elective ARCR, American Society of Anesthesiologists’ physical status I or II, age 18–75 years, and a body mass index (BMI) of 18–30 kg/m^2^. The exclusion criteria were as follows: lack of patient consent; allergy to ropivacaine; BMI >30 kg/m^2^; secondary shoulder surgery; severe bleeding disorders (such as hemophilia); block-site infections; insulin-dependent/non-dependent diabetes; neurological disorders (such as cerebrovascular disease, periodic paralysis, progressive muscular dystrophy, ankylosing muscular dystrophy, and ataxia); severe chronic obstructive pulmonary disease with forced expiratory volume <40% predicted; hepatic and renal insufficiency defined as an abnormally high level of transaminases, creatinine, or urea nitrogen in the blood; chronic pain defined as a need for >30 mg oral morphine or equivalent per day; inability to understand the visual analog scale (VAS); uncontrolled hypertension; ischemic heart disease; or communication difficulties. The data of subjects converted to open surgery intraoperatively were not recorded.

### 2.2 Blinding method

An investigator who was aware of the concentration gave the study drug to an anesthetist who performed the ISBPB. The patient and the anesthetist that performed the ISBPB were blinded to the concentration of the drug administered. An independent investigator blinded to the drug concentration evaluated the postoperative indicators within 24 h after the surgeries.

### 2.3 Study procedure

#### 2.3.1 Block administration technique

**FIGURE 1:**
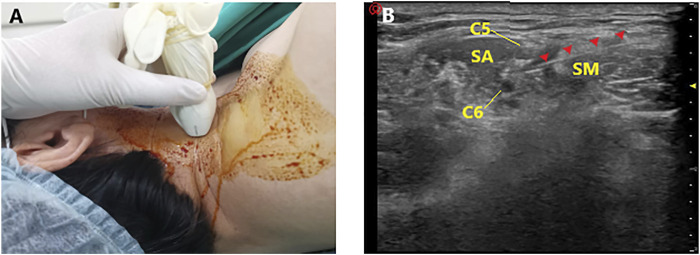
Ultrasound-guided interscalene brachial plexus block. **(A)** Placement of the ultrasound probe. **(B)** Ultrasonography of interscalene brachial plexus block (SA, scalenus anterior; SM, scalenus medius; Red arrow: ultrasound image of the needle).

Each patient was placed in the lateral position with the head facing opposite the side to be blocked. A high-frequency linear array transducer (5–12 MHz) was placed 2–3 cm above the clavicle to identify the brachial plexus in the short-axis view (Figure 1A). Under aseptic conditions, a 40-mm, 22-gauge insulated needle (UniPlex Nanoline; Pajunk, Geisingen, Germany) was advanced from lateral to medial using an in-plane method towards the point between the C5 and C6 roots, or the lateral border of the superior trunk (Franco and Williams, 2016). After positioning and negative aspiration, 10 ml of the study drug solution was injected around the C5 and C6 roots or the superior trunk (Figure 1B).

#### 2.3.2 Up-and-down method

The concentration of ropivacaine administered was determined using the small-sample UDM sequential allocation design for binary response variables (Saranteas et al., 2014). Given previous reports (Borgeat et al., 2010; Fredrickson et al., 2010; Warrender et al., 2017) and extensive clinical practice, a concentration of 0.3% of 10 ml ropivacaine (3 ml 1% ropivacaine +7 ml saline) was used in the first patient. After successful postoperative analgesia (in the initial 8 h after ISBPB, VAS score <4), the concentration of local anesthetic in the next patient was decreased by 0.05% (-0.5 ml 1% ropivacaine, diluted to 10 ml with saline). However, if the postoperative analgesia was unsuccessful, the local anesthetic concentration was increased by 0.05% (+0.5 ml 1% ropivacaine, diluted to 10 ml with saline) in the next patient. To avoid local anesthetic toxicity, all patients received <3 mg/kg of ropivacaine. No dexamethasone or dexmedetomidine was administered during the surgery to avoid adjuvant effects.

#### 2.3.3 Block evaluation

After the patient received the ropivacaine injection, the sensory and motor blocks were assessed every 5 min for up to 30 min by the anesthetist performing the ISBPB. The sensory block was assessed by the presence of a pinprick sensation in the area of the shoulder in the distribution of the brachial plexus and was classified into three grades: grade 1 = normal sensation within the nerve distribution (no block); grade 2 = dull sensation (analgesia); and grade 3 = no sensation (anesthesia). The motor block was assessed by instructing the patient to perform shoulder abduction, and the block effect was classified into three grades: grade 1 = patient reported no difference in movement from pre-block; grade 2 = patient reported reduced movement; grade 3 = no movement (complete motor block). Considering the preoperative limitation of movement due to rotator cuff injury, we defined “reduced movement” as a reduction in abduction compared to the preoperative period. Attainment of grade 2 or 3 sensory blocks and motor block within 30 min after the ISBPB was considered successful block, allowing the ARCR procedure.

#### 2.3.4 Anesthesia protocol

No subjects took any preemptive analgesics preoperatively. Before the study procedure, the patients were provided standardized instructions on how to use the patient-controlled intravenous analgesia (PCIA) pump and were trained to use the VAS.

Monitoring for non-invasive blood pressure, heart rate, and oxygen saturation was set up once the patient arrived the operation room. After the ISBPB (30 min), general anesthesia was performed for the surgery with 0.3–0.4 μg/kg sufentanil, 1–2 mg/kg propofol, and 0.7–0.8 mg/kg rocuronium for the induction of tracheal intubation. Anesthesia was maintained with sevoflurane of >0.5 minimum alveolar concentration. During the operation, the anesthesiologist added sufentanil and/or propofol intravenously as required. All patients received a prophylaxis of 1–2 mg intravenous droperidol at the end of the surgery. Routine extubation was performed after the patient was fully conscious and had achieved restored muscle strength. If the patient reported a pain score of >4 in the post-anesthesia care unit, remedial analgesia with 50 mg flurbiprofen ester was administered intravenously. Patients were taken to the ward when they were fully awake (Aldrete and Kroulik, 1970; Liu et al., 2013).

### 2.4 Postoperative pain assessment and management

After returning to the ward, all patients were treated with intravenous PCIA of 1 μg/ml sufentanil. PCIA was set at a continuous infusion rate of 3 ml/h, a bolus of 2 ml, and a lockout time of 15 min. PCIA was discontinued 48 h after surgery. The patients’ pain scores were recorded at 0, 10, 20, 30, 45 min, 1, 2, 4, 6, 8, 12, and 24 h after the surgery. Given the extreme discomfort associated with postoperative rebound pain, patients were administered a combination of oral ibuprofen (600 mg) and a bolus of PCIA for remedial analgesia if their VAS score was initially >4. Thereafter, the patients no longer received oral remedial analgesia but PCIA as required.

### 2.5 Adverse events

The adverse events considered in the study included phrenic nerve palsy, Horner’s syndrome, postoperative nausea and vomiting, local anesthetic systemic toxicity (blurred vision, hearing impairment, sleep disturbances, dizziness, muscle twitching, and arrhythmia), vascular puncture, pleural puncture, residual block, and continuous neurological deficits.

### 2.6 Statistical analysis

Appropriate measures of central tendency and dispersion or counts and percentages for categorical data were used to summarize and express patient data. In most cases, the exact sample size for Dixon’s UDM could not be determined in advance. When six crossovers (conversion from successful to unsuccessful block or vice versa) had occurred, we ceased recruiting patients (Liu et al., 2013). At least 20–40 patients were required to provide reliable estimates of the target dose in our simulation studies in anesthesia trials using Dixon’s UDM. Four statistical approaches were used to explore the MEAC, including three parametric estimates of the dose-response curve (Paul and Fisher, 2001) (linear, linear-logarithmic, and exponential regressions) and one nonparametric model (centered isotonic regression), which was only used to assume a nondecreasing dose-and-response relationship (Pace and Stylianou, 2007). Residual standard errors, a statistical tool to determine the goodness of fit, which analyzes how well a set of data points fit with the actual model, were calculated for all four statistical approaches.

## 3 Results

**FIGURE 2:**
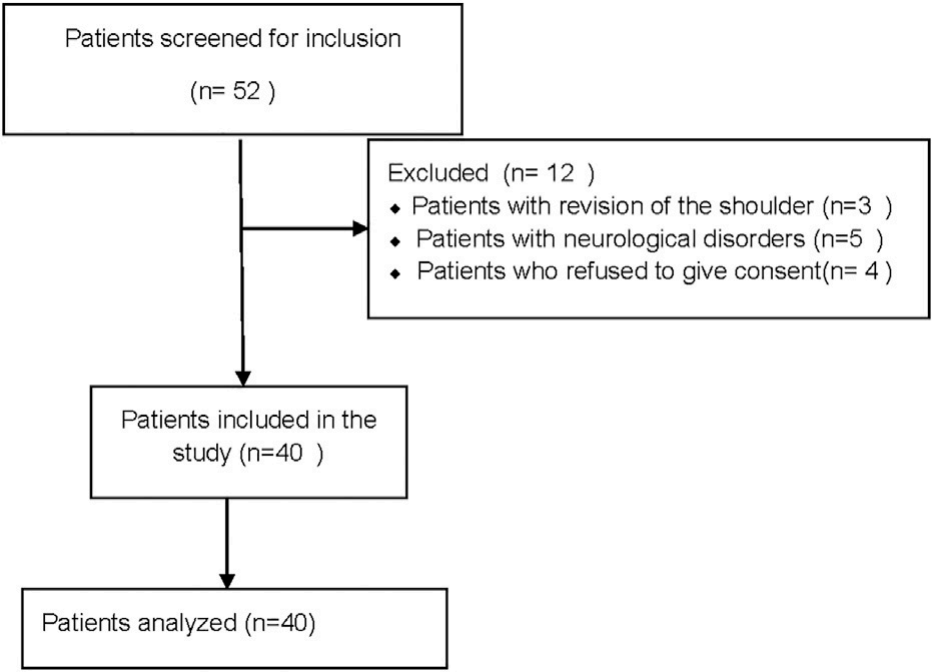
The flow of participants in the study.

**FIGURE 3:**
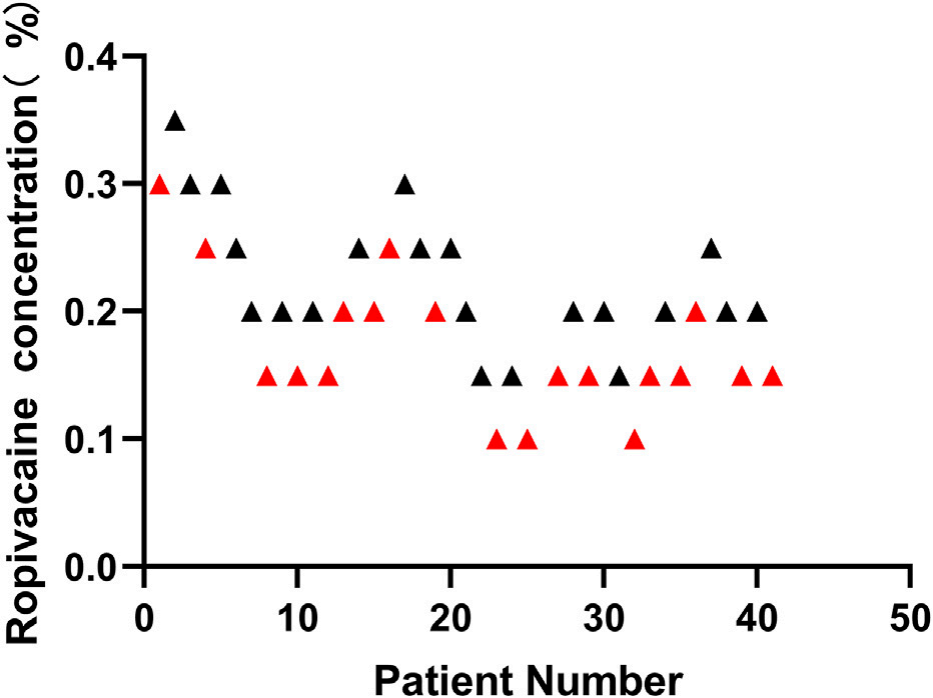
According to the Dixon and Massey up-and-down method, sequential block results of ultrasound-guided Interscalene Brachial Plexus Block using 10 ml ropivacaine. Red represents failed postoperative analgesia and black represents successful postoperative analgesia. After successful postoperative analgesia (in the initial 8 h after ISBPB, VAS score <4), the concentration of local anesthetic in the next patient was decreased by 0.05%. However, if the postoperative analgesia was unsuccessful, the local anesthetic concentration was increased by 0.05% in the next patient.

From July 2021 to August 2021, 52 patients were screened. Forty patients met the inclusion criteria and were enrolled in the study (Figure 2). None of them had a sensory block of grade 1. Most were male, their mean age was 53.8 years, and their mean BMI was 23.1 kg/m^2^ (Table 1). Figure 3 shows the ten independent UDM deflections.

**TABLE 1:** Patient characteristics.

Characteristic	Mean ± SD or No. (%)
Sex (male/female)	22/18
Age (yr)	53.8 ± 7.68
Body mass index (kg/m^2^)	23.1 ± 1.38
ASA physical status (I/II)	23/17
Duration of surgery (min)	64.4 ± 16.99
sufentanil consumption (μg)	26.4 ± 3.58
Time to 1st rescue analgesic (hr)	7.7 ± 2.44
Time to remove the laryngeal mask (min)	6.6 ± 3.55
Onset time of sensory block (min)	5.8 ± 3.33
Onset time of motor block (min)	12.9 ± 2.81
Analgesic satisfaction (1/2/3)	0/21/19

ASA, american society of anesthesiologists.

### 3.1 Median effective analgesic concentration of ropivacaine

**FIGURE 4:**
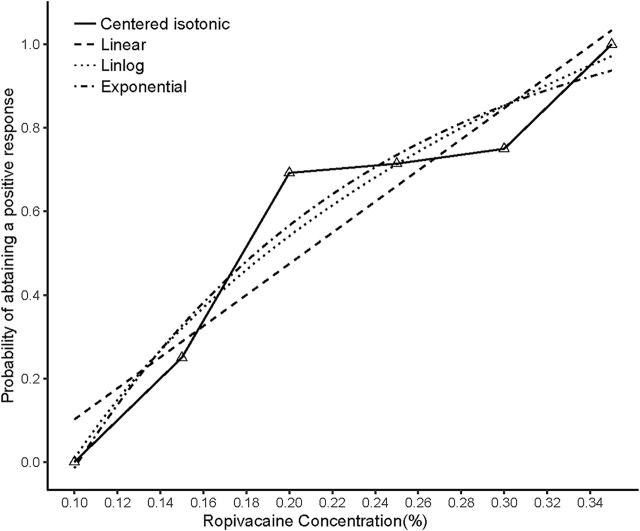
Estimated ropivacaine–Interscalene Brachial Plexus Block relationship for a given dose level and probability of successful block. Median estimators for each model are plotted. Numbered triangles represent the numbers of measurements at each ropivacaine concentration.

The sensory and motor block onset time was not significantly different between patients with successful and unsuccessful postoperative analgesia (*p* = 0.4186, *p* = 0.4554, respectively). The illustration of the sequence of successful and unsuccessful postoperative analgesia is shown in Figure 4. The linear model estimator led to an MEAC of 0.207%, the linear-logarithmic model resulted in an MEAC of 0.182%, the exponential regression yielded an MEAC of 0.196%, and the centered isotonic regression (a nonparametric method) yielded a MEAC of 0.163% (Figure 3). The 95% confidence intervals (CI) for the three parametric models (linear, linear-logarithmic, and exponential) were 0.168%–0.355%, 0.165%–0.353%, and 0.154%–0.356%, respectively (Table 2). All four models showed similar fitted probabilities within the range of the MEAC, and the 95% CIs of these models successfully covered all observed data. The results of the residual standard deviations for the goodness of fit of each model are shown in Table 2. The exponential regression had the least residual standard error (0.0990) among all models.

**TABLE 2:** The mean effective concentration and 95% confidence interval of the different models.

Model	ED50 (%)	95%CI (%)	Residual standard error
Centred isotonic			
Regression	0.163		
Linear	0.207	0.168, 0.355	0.1348
Linlog	0.182	0.165, 0.353	0.1111
Exponential	0.196	0.154, 0.356	0.0990

### 3.2 Requirement for postoperative pain and rescue analgesia

**FIGURE 5:**
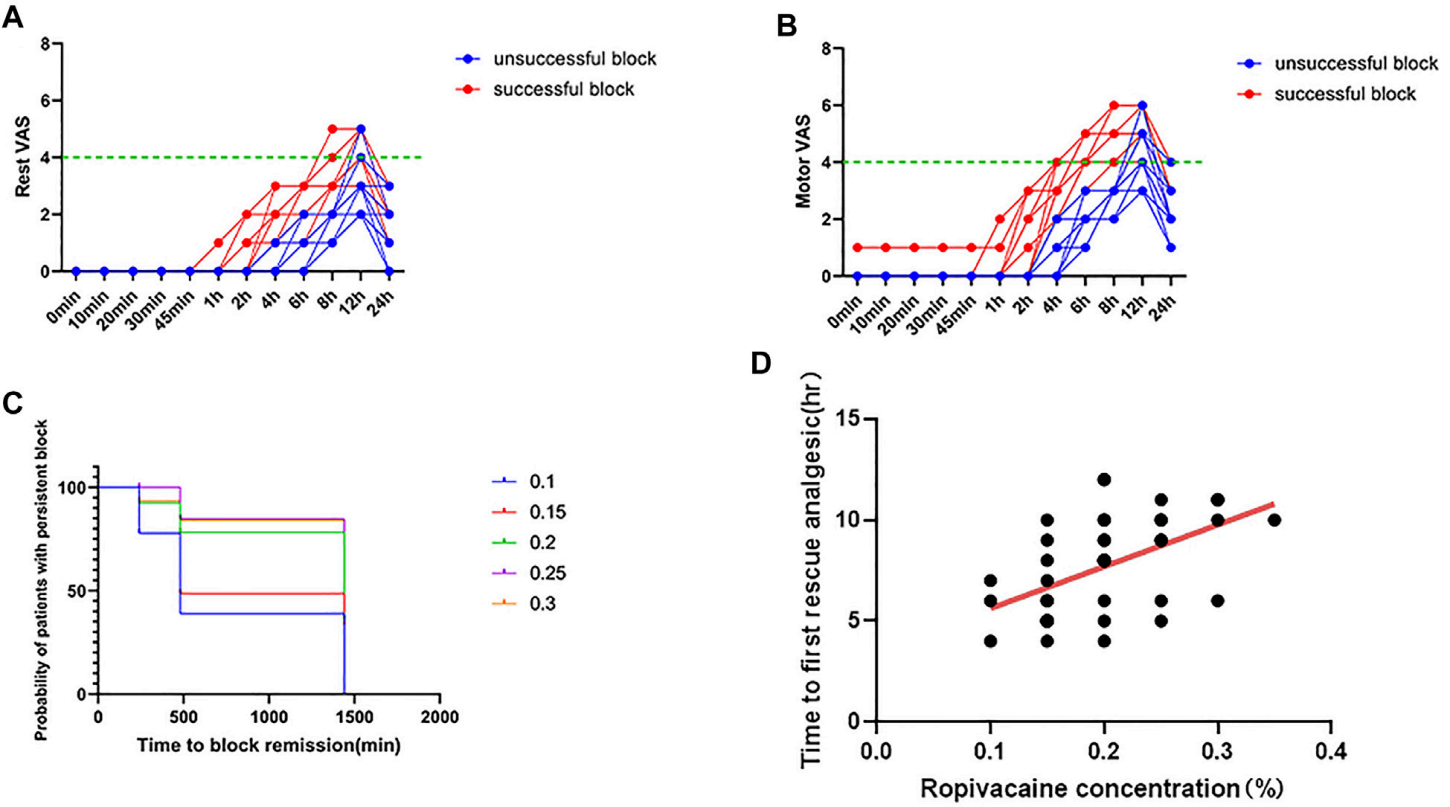
Postoperative pain scores. **(A)** Rest pain score 24 h after surgery. **(B)** Motor pain score 24 h after surgery. **(C)** Duration of the Interscalene Brachial Plexus Block with different concentrations of ropivacaine. **(D)** Correlation between ropivacaine concentration and time to first rescue analgesia in interscalene brachial plexus block.

In the study, 21 patients achieved successful postoperative analgesia. All patients with successful postoperative analgesia had a postoperative VAS score of <4 in the initial 8 h (Figure 5A and Figure 5B). The average intraoperative consumption of sufentanil (mean value ± standard deviation) was 26.4 ± 3.58 μg. The intraoperative consumption of sufentanil was not significantly different between the patients with successful and unsuccessful postoperative analgesia (*p* = 0.8191). The average time to the first rescue analgesia (mean value ± standard deviation) was 7.7 ± 2.44 h. The time to the first rescue analgesia was significantly different between the patients with successful and unsuccessful analgesia (*p <* 0.0001). The time to the first request for analgesia showed a moderate positive correlation with the local anesthetic concentration (Spearman’s r = 0.5383; the r value was significant, *p* = 0.0003) (Figure 5C and Figure 5D). The sensory and motor block onset times were not significantly different between patients with successful and unsuccessful postoperative analgesia (*p* = 0.4186, *p* = 0.4554, respectively).

### 3.3 Postoperative adverse events

No adverse effects were observed, including pneumothorax, phrenic nerve block, recurrent laryngeal nerve block, and local anesthetic systemic toxicity. Postoperative nausea and vomiting were not reported. All of the blocks wore off within 48 h after the surgeries. The above data were obtained from patients’ oral reports at the postoperative follow-up.

## 4 Discussion

This study found that the MEAC of ropivacaine was 0.196%. We chose UDM, which has the advantage of providing a simpler and more practical method of obtaining accurate MEAC than controlled studies. In our study, UDM was chosen as the concentration discovery technique rather than the continuous reassessment method, because the latter method has a model-based design that is more suitable for estimating maximum tolerated concentrations (EC95). In contrast, UDM is more suitable for MEAC calculations, with the advantages of simplicity and low sample requirements.

Ropivacaine at a concentration of 0.20%–0.75% can be used for successful ISBPB in ARCR (Zhai et al., 2016; Warrender et al., 2017). Concentrations of 0.3%–0.5% are also commonly used to administer peripheral nerve blocks in many medical centers. However, increased concentrations of ropivacaine may cause motor and sensory blockades in the distal elbow, which is detrimental to the rapid postoperative recovery of patients undergoing shoulder surgery (Krone et al., 2001). Studies on the median effective anesthetic volume of 0.75% ropivacaine for ISBPB in ARCR are now conclusive (Gautier et al., 2011; Vandepitte et al., 2013). However, no research has been published on the MEAC of ropivacaine. We designed this clinical study to investigate an appropriate concentration of ropivacaine that would provide adequate analgesia and reduce sensory and motor abnormalities at the nonsurgical site caused by high concentrations of ropivacaine in order to accelerate the patient’s recovery.

Ultrasound visualization effectively reduces the volume of local anesthetic in peripheral nerve blocks (Marhofer et al., 2010). The contemporary literature reported that low-volume (5–10 ml) local anesthetics for ISBPB could achieve the same block duration and efficacy (McHardy et al., 2020; Safa et al., 2021). Low-volume ropivacaine injections (5 ml), which are associated with a more favorable risk profile, have a less central (foraminal) and less abnormal spread compared to high-volume (20 ml) injections in ISBPB (Stundner et al., 2016). In this study, 10 ml of ropivacaine was used to cover C5 and C6, because 5 ml of ropivacaine may render the effort of finding the MEAC futile, and the generalizability of lower volume ISBPB can be limited.

Three parametric statistical (linear, linear-log, and exponential) models were used to determine the MEAC and 95% CI, and one nonparametric method (central isotope) was also used to calculate the MEAC to provide additional comparisons. Central isotope regression is a rapid and straightforward statistical method for nonparametric estimation in dose-response and dose-finding studies. It does not require a specific form of relationship and is more suitable than UDM for analyzing study data. The results showed that the MEAC values for the four models were distributed within a narrow range of 0.163%–0.196%; the 95% CIs for the three parametric models had similar probabilities of fitting within the range of MEAC, and the 95% CI for these models covered all observations within the 0.1%–0.35% concentration range.

Not all models may be appropriate for a particular study. Indeed, only one of several statistical techniques could be more suitable for the study data than others. A goodness-of-fit test can help to resolve this difficulty. After the four models were built, we calculated the standard deviation of the residuals to analyze how well a set of variables fit the actual model and to assess the strength of fit of each model. The results showed that the exponential regression model had the slightest standard deviation of residuals (0.0990), implying that this statistical model fit the study data better than the others (Figure 4).

No adverse events were reported in this study, which was attributed to the small volume of local anesthetic, accurate ultrasound visualization techniques (Kessler et al., 2015), and the experienced anesthetists. We did not monitor diaphragmatic function in the subjects, and no patient complained of dyspnea or experienced decreased oxygen saturation, but this does not exclude the possibility of diaphragmatic palsy. In patients without underlying lung disease, such paralysis is usually tolerated (Tran et al., 2017).

Most rotator cuff surgery is performed in an ambulatory surgical setting, with patients discharged home on the day of surgery (Liu et al., 2018). However, research data indicate that a minority of patients still require contact with health services after outpatient surgery due to inadequate pain management (Brix et al., 2017). Such incidents are probably more common in China than in Europe and the United States. Given the complexity of postoperative management, patients have shoulder surgery in an inpatient setting in most medical centers in China. The generalizability of our research strategy to institutions performing ambulatory surgery is limited.

There are some limitations to this study. First, UDM allows the determination of an MEAC for a clinical variable with a binary outcome in smaller sample sizes (Subramanyam et al., 2015). UDM is unreliable for calculating small or large percentiles, unlike EC95, which is a more relevant indicator for clinical applications (Pace and Stylianou, 2007). However, exploring EC95 in a small sample of 40 simulated calculations showed significant inaccuracy. When using UDM to calculate MEAC, the premise is that the dose-response relationship is a traditional S-curve, which may be incorrect. Second, the analgesic effect of peripheral nerve blocks is determined by concentration and volume, but this study only examined the concentration of a set volume of 10 ml. Subsequent studies could further explore MEAC by increasing the volume of the local anesthetic.

## Conclusion

In conclusion, the MEAC of ropivacaine in ARCR was 0.196%. In future studies, we would like to explore the appropriate volume of ropivacaine for postoperative analgesia in ARCR. In addition to finding new nerve block techniques, we majorly aim to explore methods to prolong the duration of nerve blocks.

## Data Availability

The raw data supporting the conclusion of this article will be made available by the authors, without undue reservation.

## Abbreviations

ARCR, arthroscopic rotator cuff repair; BMI, body mass index; CI, confidence interval; ISBPB, interscalene brachial plexus block; MEAC, median effective analgesic concentration; PCIA, patient-controlled intravenous analgesia; UDM, up-and-down method; VAS, visual analog scale.
